# Exploring the role of Mn^2+^ in the structure, magnetic properties, and radar absorption performance of Mn_*x*_Fe_3−*x*_O_4_–DEA/MWCNT nanocomposites

**DOI:** 10.1039/d3ra05333d

**Published:** 2023-10-09

**Authors:** Wida Puteri Agista, ST. Ulfawanti Intan Subadra, Ahmad Taufiq, Arif Hidayat, Erfan Handoko, Mudrik Alaydrus, Tahta Amrillah, Itthipon Jeerapan

**Affiliations:** a Department of Physics, Faculty of Mathematics and Natural Science, State University of Malang Jl. Semarang 5 Malang 65145 Indonesia ahmad.taufiq.fmipa@um.ac.id; b Department of Physics, Faculty of Mathematics and Natural Sciences, Universitas Negeri Jakarta Jl. Rawamangun Muka 1 Jakarta 13220 Indonesia; c Department of Electrical Engineering, Universitas Mercu Buana Jl. Meruya Selatan 1 Jakarta 11650 Indonesia; d Nanotechnology Engineering, Faculty of Advanced Technology and Multidiscipline, Universitas Airlangga Jl. Ir. Sukarno 1 Surabaya 60115 Indonesia; e Division of Physical Science, Faculty of Science, Prince of Songkla University Hat Yai Songkhla 90110 Thailand

## Abstract

Iron oxide/carbon-based nanocomposites are known as an ideal combination of magnetic–conductive materials that were recently developed in radar absorption application; one example is the Fe_3_O_4_/multiwalled carbon nanotubes (MWCNTs). In this study, we try to boost their radar absorption ability by Mn-ion doping. Mn is an appropriate Fe substitute that is predicted to alter the magnetic properties and enhance the conductivity, which are crucial to developing their radar absorption properties. Diethylamine (DEA) is also used as a capping agent to improve the size and shape of the nanocomposite. In this study, a Mn_*x*_Fe_3−*x*_O_4_–DEA/MWCNT nanocomposite is successfully prepared by the coprecipitation method using a variation of *x* = 0, 0.25, 0.5, 0.75, and 1. We found that the sample's magnetic saturation (*M*_s_) decreases, while the reflection loss (RL) increases with increasing the molar fraction of Mn. The enhancement of the radar wave absorption in the sample is dominated by dielectric losses due to the increase of electrical conductivity and interfacial polarization with the addition of Mn in the nanocomposites. We believe that our finding could shed light on the role of doping elements to develop the radar absorption properties, and further pave the way for the real implementation of iron oxides/graphene-based nanocomposite as radar-absorbing materials (RAMs).

## Abstract 2

Iron oxide/carbon-based nanocomposites are known as an ideal combination of magnetic–conductive materials that were recently developed in radar absorption application; one example is the Fe_3_O_4_/multiwalled carbon nanotubes (MWCNTs).

## Graphical abstract

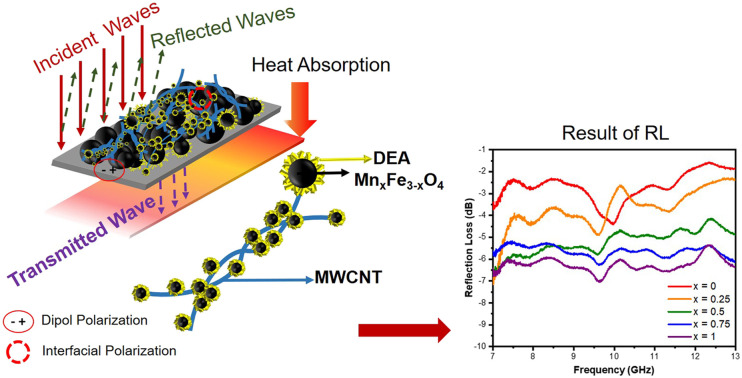

## 1. Introduction

Recently, the escalating growth of communication technology, particularly wireless communication, has led to a significant rise in electromagnetic pollution, emerging as a critical concern.^1^ Electromagnetic pollution damages electronic devices, and has a detrimental effect on human health.^2,3^ Therefore, researchers are currently working on the development of microwave-absorbing materials, particularly radar-absorbing materials (RAMs). Apart from addressing the issue of electromagnetic pollution, RAMs have potential applications in the military sector to conceal military defense equipment, such as fighter planes and tanks, from adversaries.^4^ RAMs attenuate the energy of electromagnetic waves by converting the waves into heat energy through magnetic and dielectric losses. Nevertheless, the primary challenge in RAM design lies in the selection of materials. To maximize their capability to absorb electromagnetic waves, a better understanding of how to engineer materials composed of magnetic and dielectric elements is required.

Fe_3_O_4_ nanoparticles (NPs) have garnered attention as potential microwave absorbents^5,6^ owing to their unique features, including outstanding magnetic properties,^7,8^ great permeability, excellent chemical and thermal stability,^9^ high biocompatibility, and nontoxicity.^10^ Despite these superiorities, Fe_3_O_4_ still has relatively low absorption^8^ and deficient dielectric properties. Fe_3_O_4_ also tends to agglomerate;^11^ therefore, the use of capping agents, such as PEG and PVP, as templates is essential.^12–14^ However, they fail to generate NPs with uniform size and shape. Diethylamine (DEA) is one of the capping agents serving as a soft template for improving the size and shape of NPs to minimize agglomeration, which is also important to boost the absorption ability.^15,16^

The performance of Fe_3_O_4_ NPs in radar absorption can be improved through compositing and substitution with other materials. Recently, carbon nanotubes (CNTs) have gained attention as composite materials with Fe_3_O_4_ NPs owing to their dielectric, mechanical, electrical, and thermal properties and extensive surface area.^17^ Multiwalled CNTs (MWCNTs) are one of the CNTs commonly composited with Fe_3_O_4_ NPs for numerous applications, including RAMs.^18^ A combination of carbon and magnetic materials is considered as an effective strategy to obtain high-performance RAMs.^19^ For RAM application, MWCNT offers excellent conductivity.^20^ Combining MWCNT with Fe_3_O_4_ enhances the absorbing intensity and bandwidth and reduces RAM density, which benefits the performance of the RAM.^21^ Fe_3_O_4_ as a magnetic component also could enrich the electromagnetic wave dissipation mechanism, which is important in wave absorption ability.^22^ Nevertheless, although the iron oxides/graphene-based nanocomposite is known as an ideal combination of magnetic–conducting materials that was recently developed in radar absorption applications, further development is required. Various attempts had been carried out, such as element/ion substitution, shape or morphology modification, and compositing strategies. Among them, we believe that element/ion substitution could substantially develop the RAM. Thus, an in-depth exploration of this strategy is very important.

In this study, we performed Mn^2+^ ion substitution to improve the radar absorption ability and performance of Fe_3_O_4_–DEA/MWCNT. Mn^2+^ ion substitution can alter the magnetic properties of iron oxides since it has the appropriate atomic radii with Fe.^23^ Magnetic properties are one of the vital parameters for Fe_3_O_4_ NP application in radar absorption application.^24^ Mn ion substitution can also increase the conductivity of a material, including Fe_3_O_4_, which affects the dielectric loss of the RAM.^23,24^ This study aims to identify the effects of Mn^2+^ ion substitution on the structure, morphology, and magnetic properties of Fe_3_O_4_–DEA/MWCNT nanocomposites. An in-depth study of the effect of Mn substitution is very important to pave the way for the real implementation of Fe_3_O_4_–DEA/MWCNT and other types of iron oxides/graphene-based nanocomposites as RAMs.

## 2. Experimental method

### 2.1. Synthesis of Mn_*x*_Fe_3−*x*_O_4_–DEA/MWCNT nanocomposites

Iron sand was used as the central precursor in the Mn_*x*_Fe_3−*x*_O_4_ preparation. MnCl_2_·6H_2_O, DEA, HCl (12 M, 99.9%), NH_4_OH (6.5 M, 999%), and HNO_3_ (37%) were purchased from Merck. MWCNTs were obtained from Sigma-Aldrich, and distilled water was acquired from Pro Analysis. MWCNTs were functionalized as previously described.^25^ First, 1 g of MWCNTs was mixed with 100 mL of HNO_3_, and sonicated for 2 h at 40 kHz and 50 °C. The solution was filtered, washed using distilled water until pH 7, and then dried in an oven at 100 °C for 5 h to produce functionalized MWCNT powder (F-MWCNT). The synthesis of nanocomposites began with the production of Mn_*x*_Fe_3−*x*_O_4_. First, iron sand was separated using a permanent magnet to select the magnetic powder with high purity.^26^ Then, 20 g of magnetic powder was reacted with 58 mL of HCl, followed by stirring for 30 min at room temperature to produce FeCl_2_ and FeCl_3_ solutions. MnCl_2_·6H_2_O with Mn_*x*_ fraction variations of *x* = 0, 0.25, 0.5, 0.75, 1 was then added. We controlled the amount of molar fraction *x* with the calculated Mol divided with Mr, and reaction of Mn_*x*_Fe_3−*x*_O_4_ followed eqn (1).

1 *x*Mn^2+^ + (1 − *x*)Fe^2+^ + 2Fe^3+^ + 8OH^−^ → Mn_*x*_Fe_3−*x*_O_4_ + 4H_2_O

The solution was then mixed for 20 min, and titrated with 6 mL of DEA solution previously dissolved into 9 mL of distilled water. The titration products were combined with 0.1 g of F-MWCNT while stirring at room temperature. A total of 19 mL of NH_4_OH was titrated until a black precipitate was obtained. The precipitate was washed using distilled water until pH 7, and then dried at 100 °C to attain a sample of the Mn_*x*_Fe_3−*x*_O_4_–DEA/MWCNT powder.

### 2.2. Characterizations

The structure, phase, crystallite size, and lattice parameter of the Mn_*x*_Fe_3−*x*_O_4_–DEA/MWCNT nanocomposites were investigated by X-ray diffraction (XRD) using X'Pert Pro test, Cu-Kα 1.540 Å Panalytical Merck with Cu-Kα 1.54060 Å radiation beam. The morphology and components of the nanocomposites were determined by scanning electron microscopy (SEM) combination of carb characterization type FEI, Inspect-S50. The functional groups within the sample were investigated using Fourier transform infrared (FTIR) spectroscopy type IRPrestige-21. The magnetic properties of the nanocomposites were characterized using a vibrating sample magnetometer (VSM) type PPMS VersaLab with a magnetic field ranging from −3 to 3 Tesla. Lastly, the radar absorption performance of the nanocomposites was characterized using vector network analyzer (VNA) type Rohde-Schwarz ZVA 67 to determine the complex permittivity, complex permeability, and reflection loss (RL).

## 3. Results and discussion

**Fig. 1:**
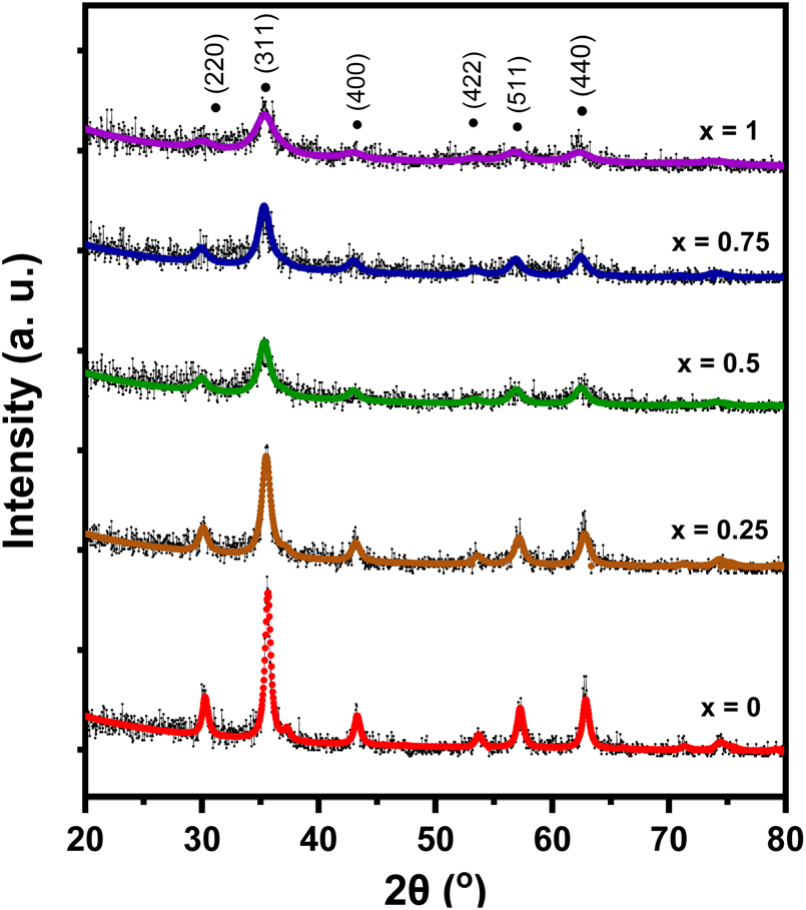
Diffraction pattern of Mn_*x*_Fe_3−*x*_O_4_–DEA/MWCNT with different compositions of Mn substitution.

The XRD pattern of Mn_*x*_Fe_3−*x*_O_4_–DEA/MWCNT is presented in Fig. 1. Diffraction peaks with *x* = 0 code (Fig. 1) were detected at 2*θ* = 30.21°, 35.57°, 43.13°, 53.65°, 57.13°, and 62.69°. This diffraction pattern is similar to that reported for Fe_3_O_4_–DEA.^15^ The absence of new peaks following DEA addition suggests the successful utilization of DEA as a surfactant.^16^ Furthermore, the lack of MWCNT diffraction peaks at 2*θ* = 26° is caused by the lower mass of MWCNT relative to that of Mn_*x*_Fe_3−*x*_O_4_ at a ratio of 1 : 30 for MWCNT : Mn_*x*_Fe_3−*x*_O_4_. The diffraction patterns of all samples also indicate that the addition of Mn as a dopant (*x* = 0.25–1) generates the same pattern as that of the *x* = 0 sample. Therefore, our results showed that Mn addition causes no new peak or phase, signifying that Mn has successfully penetrated Fe_3_O_4_. Li *et al.* demonstrated that the absence of new peaks on Mn_*x*_Fe_3−*x*_O_4_ indicates that Mn^2+^ successfully enters Mn_*x*_Fe_3−*x*_O_4_, replaces Fe^3+^, and does not form MnO_2_ deposits on the Fe_3_O_4_ surface.^27^

**Fig. 2:**
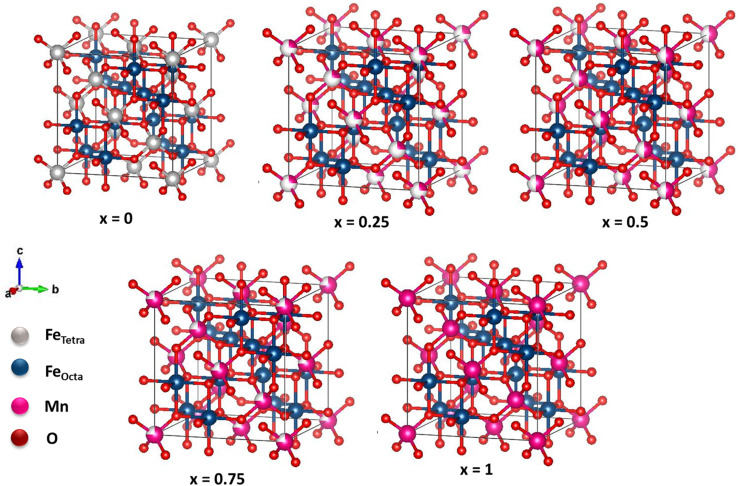
Possible crystal structure of Mn_*x*_Fe_3−*x*_O_4_ with different compositions of Mn substitution.

We performed a quantitative analysis by comparing the obtained data with the ICSD database number 30860. The results showed that the observed peaks are identical with the Miller index of (220), (311), (400), (422), (511), and (440), with the highest peak observed at ∼2*θ* = 35.5° on the *hkl* (311). This finding indicates the single phase of the sample with cubic spinel structure and the space group of *Fd*3*m*. The diffraction patterns at *hkl* (220) and (311) demonstrate a shift towards smaller 2*θ* values with Mn substitution. This shift is associated with the increase in the sample's lattice parameters (8.361–8.429 Å) due to Mn substitution, as summarized in Table 1. The observed increase in lattice parameter is attributed to the effects of ionic size because Mn^2+^ (0.81 Å) possesses a larger ionic radius compared to Fe^3+^ (0.77 Å).^28^ Therefore, Mn substitution on Fe_3_O_4_ drives the expansion of cell units, as proven by the expanded sample's crystal volume shown in Table 1 and Fig. 2. This finding also signifies that some Fe^3+^ ions on the tetrahedral structure have been substituted by Mn^2+^ ions.^24^ Our results are similar to those of a previous study reporting an increase in lattice parameters from 8.372 Å to 8.474 Å with Mn^2+^ substitution on *x* = 0.25 to *x* = 1 variations.^29^

**Table 1:** Crystallite size, lattice parameter, and crystal volume of Mn_*x*_Fe_3−*x*_O_4_–DEA/MWCNT

Samples	Crystallite size (nm)	Lattice parameter (Å)	Crystal volume (Å^3^)
*x* = 0	17.0 ± 0.2	8.361 ± 0.003	584.5 ± 0.3
*x* = 0.25	11.0 ± 0.3	8.385 ± 0.001	589.5 ± 0.2
*x* = 0.5	8.0 ± 0.1	8.404 ± 0.003	593.6 ± 0.1
*x* = 0.75	7.9 ± 0.2	8.409 ± 0.002	594.6 ± 0.2
*x* = 1	4.5 ± 0.3	8.419 ± 0.002	596.7 ± 0.5

The crystal structure of Mn_*x*_Fe_3−*x*_O_4_ is illustrated in Fig. 2. The *x* = 0 sample structure has an inverse cubic spinel structure with eight Fe^3+^ ions in the tetrahedral site, and eight Fe^2+^ and Fe^3+^ ions in the octahedral site. The addition of Mn^2+^ has enlarged the cubic spinel crystal structure compared to that of the sample *x* = 0, signifying the expansion of the cell unit. In samples with *x* = 0.25, 0.5, and 0.75, Mn^2+^ ions replace some Fe^3+^ ions on the tetrahedral site. In sample *x* = 1, all Fe^3+^ ions are substituted by Mn^2+^ ions. Four and six O ions are observed surrounding the Fe^2+^, Fe^3+^, and Mn^2+^ ions on the tetrahedral and octahedral sites, respectively.

**Fig. 3:**
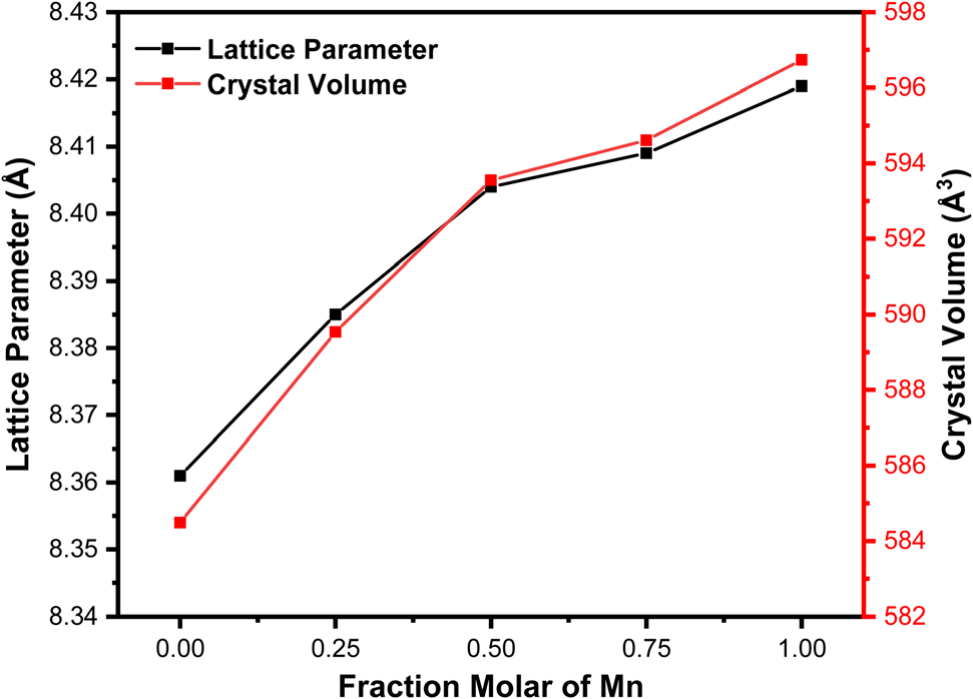
Relationship of the Mn molar fraction (*x*) with the lattice parameter and crystal volume.

Fig. 3 displays the graph depicting variations in the lattice parameters and Mn molar fraction variations. The Mn molar fraction demonstrates an increasing trend with two distinct slopes: one for molar fractions ranging from 0 to 0.5, and another for molar fractions from 0.5 to 1. This observed trend closely resembles the increase in lattice parameters reported in a previous study of about ∼8.4 Å.^30^ Additionally, this trend is consistent with the Mössbauer results, which indicate that Mn^2+^ ions replace Fe^3+^ ions on the tetrahedral sites. This finding is supported by the Mössbauer spectroscopy, which shows that the iron ions on the MnFe_2_O_4_ system are in the 3+ state, allowing the substitution of Mn^2+^ to be written as (Fe_*x*−1_^2+^Fe_*x*+1_^3+^)_octa_(Mn_*x*_^2+^Fe_*x*−1_^3+^)_tetra_.

The crystallite size of the Mn_*x*_Fe_3−*x*_O_4_–DEA/MWCNT nanocomposites decreases with the increase in Mn substitution, which is consistent with previous results.^23^ The reduced crystallite size is caused by the addition of DEA as the controlling and reducing agent for the crystallite size. Taufiq *et al.* similarly reported that all synthesized Fe_3_O_4_ samples with the DEA template present a nanometric particle size, which decreases upon the addition of DEA at high concentrations.^16^ By using DEA, the particle size of the nanocomposite is easy to control. Thus, by controlling the particle size, we also can tune the absorbance ability of the nanocomposite.^19^

**Fig. 4:**
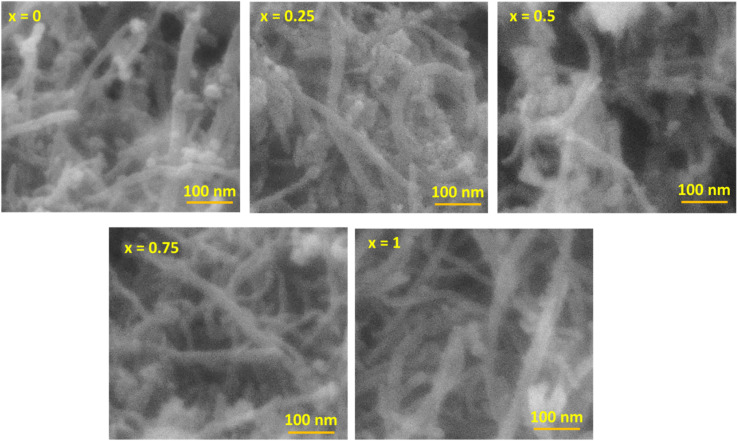
Morphology of Mn_*x*_Fe_3−*x*_O_4_–DEA/MWCNT with *x* = 0.25–1 at 100 000× magnification.

**Fig. 5:**
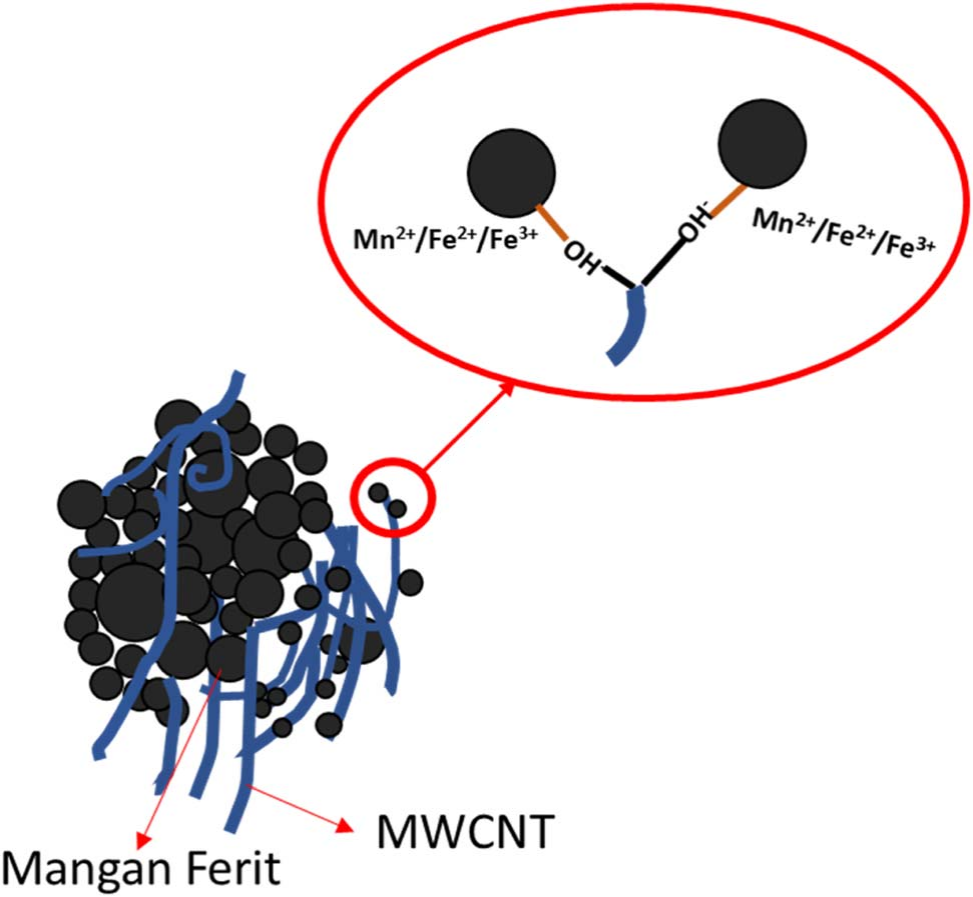
The illustration of Mn_*x*_Fe_3−*x*_O_4_–DEA/MWCNT with *x* = 0.25–1.

The morphology of the Mn_*x*_Fe_3−*x*_O_4_–DEA/MWCNT nanocomposites was assessed by SEM characterization. The SEM image with 100 000× magnification is presented in Fig. 4. The Mn_*x*_Fe_3−*x*_O_4_ cluster is in the form of a chunk, and the MWCNTs exhibit a cylindrical shape. The MWCNTs in the cylinder form a shroud and bind to the spherical Mn_*x*_Fe_3−*x*_O_4_ chunk. The bond arises due to MWCNT functionalization, which introduces OH-functional groups on the MWCNT surface. These groups interact with the positive charge of Mn_*x*_Fe_3−*x*_O_4_, as depicted in Fig. 5. Theoretically, the positive ions of Fe_3_O_4_, such as Mn^2+^, Fe^2+^, or Fe^3+^, form bonds with the negative ions and the charged carboxyl group on the MWCNT surface, facilitated by the MWCNT functionalization using HNO_3_. The presence of the carboxyl group and damage in certain parts of MWCNT facilitates its binding to other compounds, in this case, Mn_*x*_Fe_3−*x*_O_4_–DEA.^18^

The carboxyl group with a negative charge will interact with Mn_*x*_Fe_3−*x*_O_4_ having positive ions (Mn^2+^, Fe^2+^, and Fe^3+^), which initiate an electrostatic attraction and increase the structural stability of the nanocomposites.^31,32^ Due to this fact, Mn_*x*_Fe_3−*x*_O_4_ is strongly bonded to the surface of MWCNT. MWCNT also serves as a barrier to suppress the aggregation tendency of Mn_*x*_Fe_3−*x*_O_4_, and thus further increases the structural stability.^33^ In addition, the morphology of MWCNTs that form a long tube shape tends to have better conductivity because they can form a more continuous conductive path. This agrees with previous reports indicating that RAMs with tubular shape and considerable anisotropy can form three-dimensional conduction networks to enhance its conductivity.^34^ This condition favors wave propagation through the material and its attenuation. Thus, an excellent wave attenuation in this proposed RAM could be induced due to the excellent interface between CNTs and Mn_*x*_Fe_3−*x*_O_4_, which creates an interface polarization.^35^ It is important to note that the interface polarization could induce excellent dielectric loss capacity for microwave absorption of the nanocomposite.^34^ Controlling the shapes and morphology of RAMs is important because the microwave absorption ability also depends on the degree of density, weight and dispersion of RAMs itself.^36^ The morphology of the nanocomposites after Mn addition, as depicted in Fig. 4, closely resembles the nanocomposites with a molar fraction of 0. Moreover, we observed a decrease in agglomeration with Mn addition. On the other hand, the further reduction of agglomeration upon inclusion of DEA was also indicated.^18,37^

**Fig. 6:**
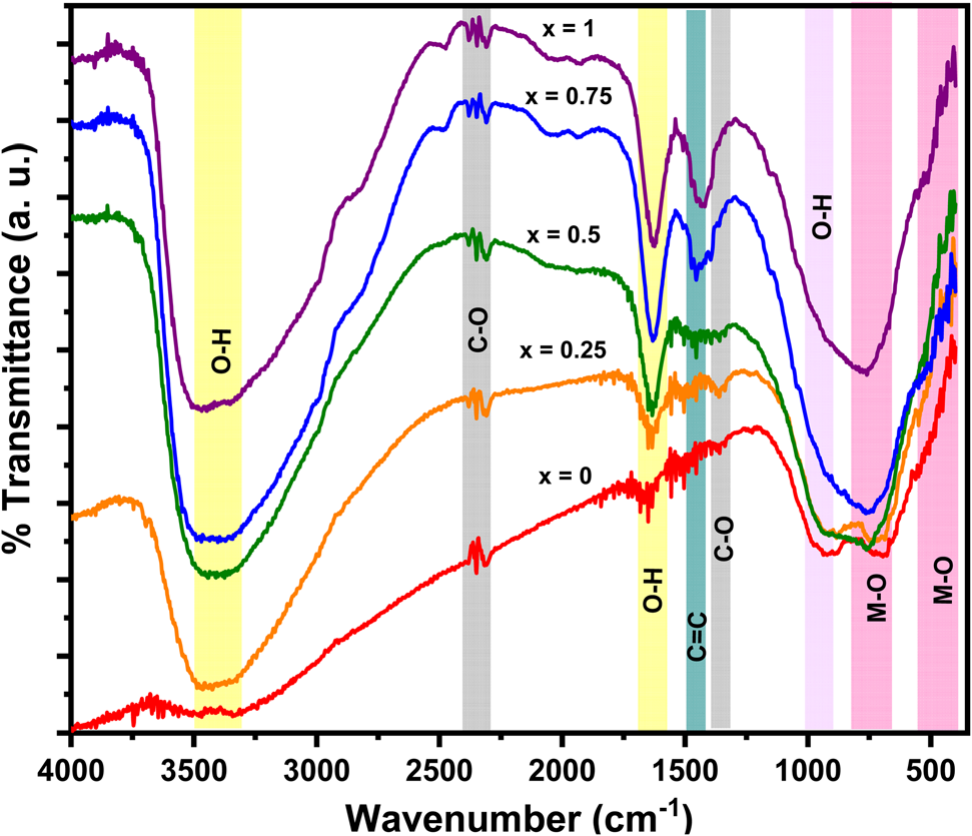
FTIR spectrum for the Mn_*x*_Fe_3−*x*_O_4_–DEA/MWCNT nanocomposites with different compositions of Mn substitution.

Fig. 6 shows the FTIR spectrum of the Mn_*x*_Fe_3−*x*_O_4_ nanocomposites. At 3491–3290 cm^−1^, we identified a widened O–H vibration, possibly induced by the –OH groups on the surface areas. This functional group facilitates the combination of Mn–Fe_2_O_4_ with MWCNT.^15,38^ Our obtained vibrations are similar to the vibrational peak of O–H reported in previous studies, specifically those at 3321,^25^ 3420,^38^ and 3430 cm^−1^.^15^ We also detected the C–O functional groups at 2325 and 1359 cm^−1^ that are possibly from the CO_2_ atmosphere^15^ and identified the C=C functional group at 1438 cm^−1^, characterizing the graphite bonds compiled in the MWCNT. No functional groups of DEA are found, especially N–H, C–N, and N–H at 733, 1143, and 3288 cm^−1^, respectively. This finding suggests that the DEA used in nanoparticle preparation has not evaporated. The functional groups of metal oxide (M–O), namely, Fe–O and Mn–O, are detected at 412 and 673 cm^−1^. These two vibrations indicate that the manganese ferrite has a cubic spinel structure at the octahedral and tetrahedral sites. This finding is in line with a previous study reporting vibration peaks in the range of 430–482 cm^−1^ and 433–573 cm^−1^.^39^ As illustrated in Fig. 6, the absorbance intensity between 600 and 700 cm^−1^ increases with further addition of Mn molar fraction. This finding confirms the success of the Mn^2+^ ions in substituting Fe^3+^ ions in the tetrahedral sites. In addition, the increasing concentration of Mn leads to a shift of absorbance peaks towards lower wavenumbers. This phenomenon is correlated with the increase in the distance between two atoms, which occurs due to the increase in lattice parameters.

**Fig. 7:**
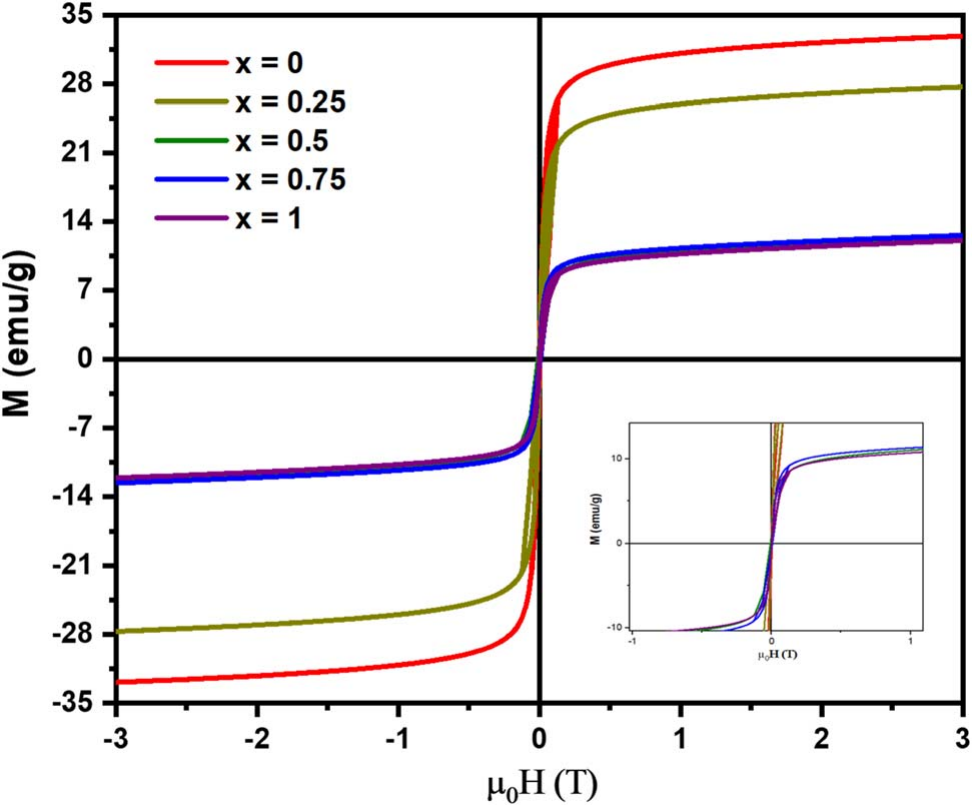
*M*–*H* hysteresis curves of the Mn_*x*_Fe_3−*x*_O_4_–DEA/MWCNT nanocomposites.

Fig. 7 shows the *M*–*H* hysteresis curve from the VSM analysis of the Mn_*x*_Fe_3−*x*_O_4_–DEA/MWCNT nanocomposites. All samples show superparamagnetic features as indicated by the obtained S curve. The attained hysteresis curve was then analyzed using the Langevin method with susceptibility,^40^ and the results are summarized in Table 2. The remanent magnetization and coercivity field are close to 0, characterizing the superparamagnetic components.^30^ The highest magnetization of 32.32 ± 0.03 emu g^−1^ is observed for Mn_*x*_Fe_3−*x*_O_4_–DEA/MWCNT at *x* = 0. This value is lower than the previously reported magnetization of Fe_3_O_4_–DEA at 35.76 emu g^−1^. Therefore, the obtained values can be correlated with the addition of MWCNT (nonmagnetic), *i.e.*, MWCNT lowers the magnetic fraction of the samples and affects the *M*_s_ value. In our previous study, we uncovered that Fe_3_O_4_/MWCNT has *M*_s_ of 12.64 and 21.15 emu g^−1^, which are lower than the magnetization values obtained in the present work. The addition of DEA to the nanocomposites also affects the sample's magnetization. The magnetization values also decrease following the increase in the Mn molar fraction substituted for Fe_3_O_4_. This decrease can also be attributed to the low crystallite value, as demonstrated in Table 1. Nanosized particles typically have a single domain and exhibit paramagnetic behavior, resulting in an increased spin disorder on the nanoparticle's surface, which subsequently reduces the magnetic moment.^41^

**Table 2:** Magnetization saturation, remanent magnetization, coercivity field, and susceptibility of the Mn_*x*_Fe_3−*x*_O_4_–DEA/MWCNT

Samples	*M*_s_ (emu g^−1^)	*M*_r_ (emu g^−1^)	*H*_c_ (T)	*χ*
*x* = 0	32.32 ± 0.03	−0.013 ± 0.012	0.520 ± 0.001	0.81 ± 0.02
*x* = 0.25	27.12 ± 0.03	−0.012 ± 0.011	0.510 ± 0.003	0.84 ± 0.02
*x* = 0.5	12.07 ± 0.02	−0.010 ± 0.002	0.066 ± 0.015	0.65 ± 0.01
*x* = 0.75	11.91 ± 0.02	−0.005 ± 0.001	0.052 ± 0.001	0.78 ± 0.01
*x* = 1	11.51 ± 0.02	−0.011 ± 0.004	0.010 ± 0.002	0.71 ± 0.01

**Fig. 8:**
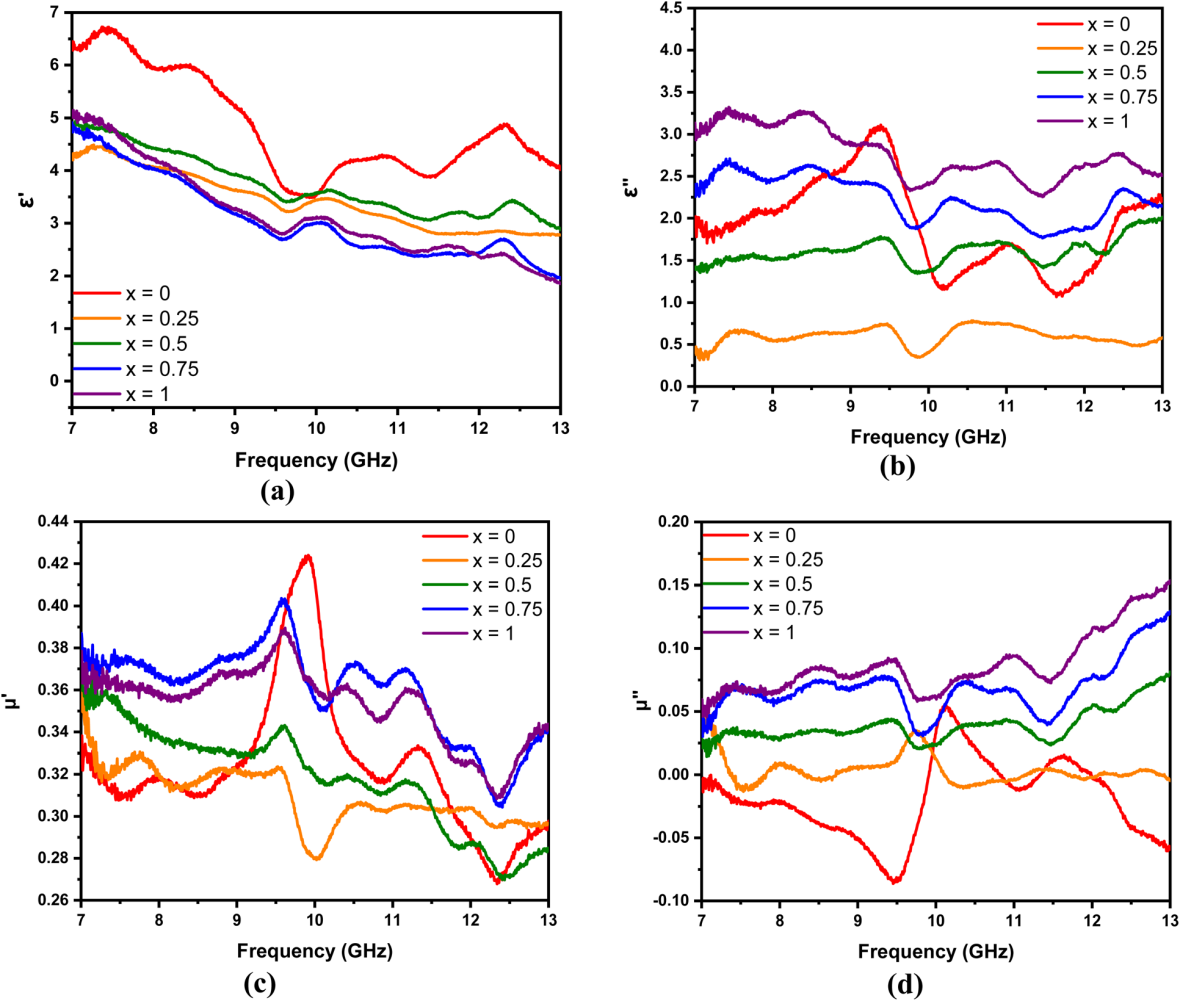
(a)–(c) Real and (b)–(d) imaginary parts of the permittivity and permeability of Mn_*x*_Fe_3−*x*_O_4_–DEA/MWCNT with different compositions of Mn substitution, respectively.

The real and imaginary parameters of the electrical permittivity (real permittivity (*ε*′) and imaginary permittivity (*ε*′′)) and the magnetic permeability (real permeability (*μ*′) and imaginary permeability (*μ*′′)) shown in Fig. 8 are parameters used to determine the absorption mechanism of the Mn_*x*_Fe_3−*x*_O_4_–DEA/MWCNT nanocomposites for radar waves. *ε*′ and *μ*′ represent the ability to store electric and magnetic energy, respectively; and *ε*′′ and *μ*′′ are related to the dissipation and loss of electric and magnetic energy, respectively. As illustrated in Fig. 8(a), which is the *ε*′ curve, the real permittivity value decreases from 6.4 to 4.2 when the molar fraction of Mn increases. Meanwhile, the *ε*′′ value tends to be greater after Mn addition, which can be explained by eqn (2).2
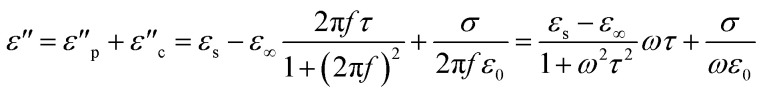


According to eqn (2), an increase in *ε*′′ is related to electrical conductivity. Mn substitution increases the conductivity value of a material due to the production of holes during charge transfer.^42^ An increase in electrical conductivity also increases the loss and dissipation ability of the electric part, as indicated by the *ε*′′ value of the nanocomposites with *x* = 1 greater than *x* = 0. In addition to electrical conductivity, the *ε*′′ value is related to interfacial polarization, which is marked by several resonance peaks around the frequencies of 7.5, 8.5, 9.5, 11, and 12.5 GHz. These peaks are related to the relaxation stage when interfacial polarization occurs.^2^

The *μ*′ and *μ*′′ of Mn_*x*_Fe_3−*x*_O_4_–DEA/MWCNT are shown in Fig. 8(c) and (d), respectively. The *μ*′ of all samples tends to decrease gradually, and the *μ*′′ increases with the frequency. Fluctuations are observed in the *μ*′ and *μ*′′ curves, especially at frequencies above 9 GHz. The fluctuation peaks represent the resonance peaks, where the absorbance peaks at low frequencies originate from the wall resonance domain, and the peaks at high frequencies correspond to natural resonance. Furthermore, the *μ*′′ of the sample with Mn addition is greater than that of the sample without Mn substitution. This finding indicates that the magnetic loss of the nanocomposites increases with the molar fraction of Mn. The *μ*′′ in the sample *x* = 0 shows a negative value at several frequencies, implying that the magnetic energy from this sample is directly emitted without being absorbed at that frequency.^43^3
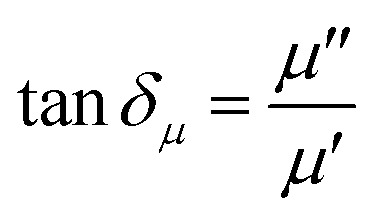
4
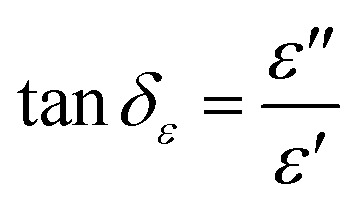


**Fig. 9:**
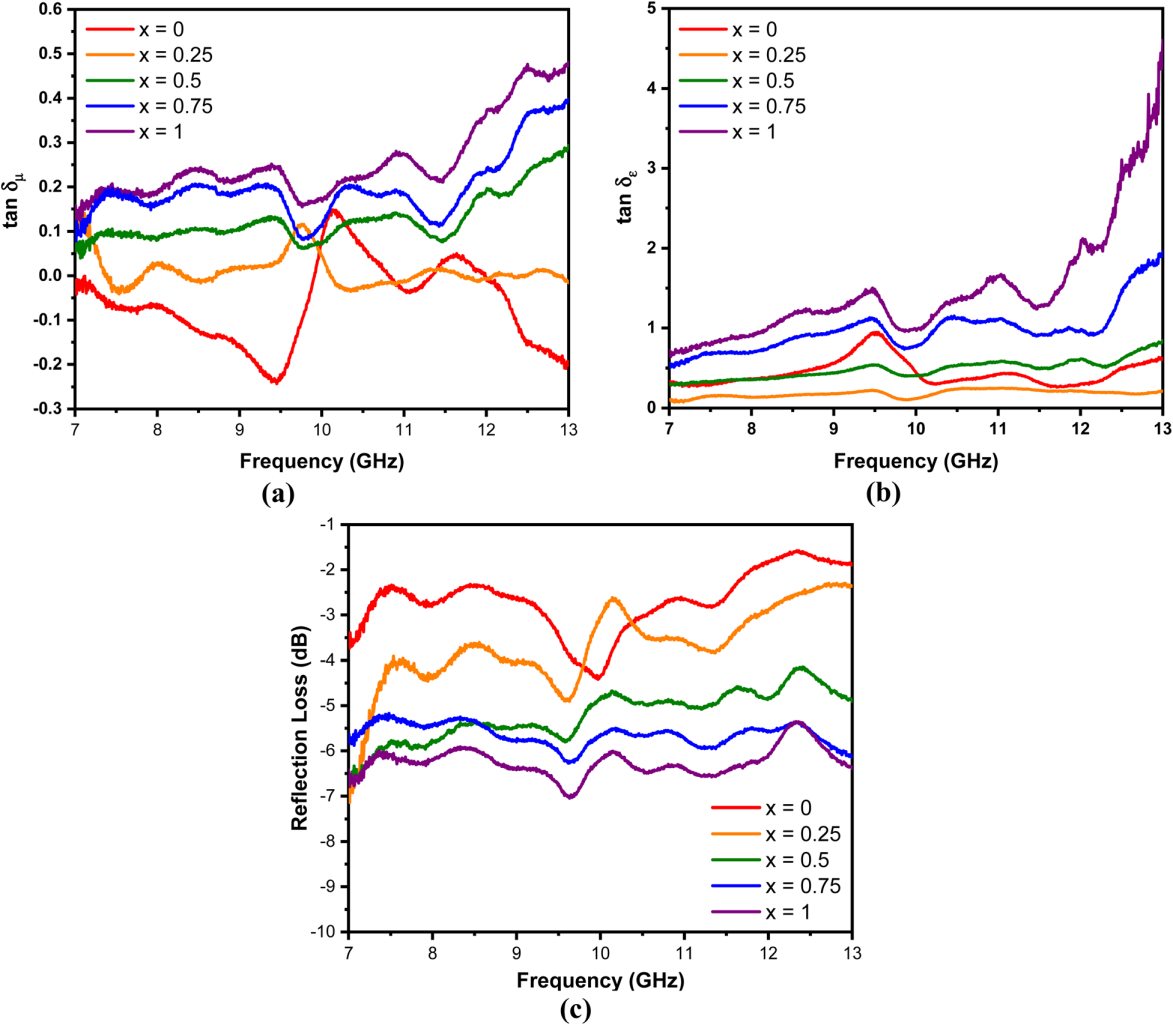
(a) tan *δ*_*μ*_, (b) tan *δ*_*ε*_, and (c) RL of Mn_*x*_Fe_3−*x*_O_4_–DEA/MWCNT with different compositions of Mn substitution.

In general, the absorption capability of radar waves is described by magnetic loss and dielectric loss, which are represented by tan *δ*_*μ*_ and tan *δ*_*ε*_, respectively. tan *δ*_*μ*_ and tan *δ*_*ε*_ describe the loss capacity of the radar absorption materials, and can be calculated using eqn (3) and (4), respectively. Fig. 9 shows the relationship of tan *δ*_*μ*_ and tan *δ*_*ε*_ with frequency. Comparison between Fig. 9(a) and (b) shows that the tan *δ*_*ε*_ value is higher than the tan *δ*_*μ*_ value. These results explain that dielectric loss is the main contributor to the absorption of radar waves by the Mn_*x*_Fe_3−*x*_O_4_–DEA/MWCNT nanocomposites.^44^

The performance of the Mn_*x*_Fe_3−*x*_O_4_–DEA/MWCNT nanocomposites for the absorption of radar waves is shown as a graph of the relationship between the reflection loss (RL) and frequency depicted in Fig. 9(c). The RL values of Mn_*x*_Fe_3−*x*_O_4_–DEA/MWCNT for *x* = 0–1 are −4.39, −4.85, −5.77, −6.26, and −7.03 dB. The increase in RL is associated with a decrease in particle size as the molar fraction of Mn increases in the nanocomposites. Nanocomposites with a small size have a large proportion of surface atoms, which can be magnetized and polarized under an external electromagnetic field. This situation allows the radar wave energy to be converted into heat, resulting in high RL values.^45^ The highest RL of −7.03 dB is recorded for the sample with *x* = 1, which is consistent with the highest tan *δ*_*μ*_ and tan *δ*_*ε*_ values (magnetic and dielectric loss) for this sample. In addition, *x* = 1 has a smaller crystallite size than other samples (Table 1). A low particle size is associated with a high dipole polarization that induces dielectric loss.^46^ In addition, the particle sizes also affect the degree of density, weight, and dispersion of nanocomposites, which further affect their absorption ability.^36^

## 4. Conclusion

Mn_*x*_Fe_3−*x*_O_4_–DEA/MWCNT nanocomposites have been successfully synthesized by the coprecipitation method having Mn fraction variations of *x* = 0, 0.25, 0.5, 0.75, 1. The crystallite size decreases from 17 to 4.5 nm with the increase in substituted Mn substitution. The morphology of the nanocomposites contains spherical shapes and chunks of Mn_*x*_Fe_3−*x*_O_4_, as well as tubular shapes of the MWCNT, indicating they are physically contacted. All of the synthesized nanocomposites exhibit superparamagnetism with the tendency of decreasing saturation magnetization at around 32.32 ± 0.03 to 11.51 ± 0.02 emu g^−1^ due to Mn addition, which presumably enhances the spin disorder on the nanocomposite's surface. Furthermore, the performance of the nanocomposites in absorbing radar waves is shown by the RL value that increases from −4.39 to −7.03 dB with the increase in the molar fraction of Mn. It shows that the radar absorption performance of the Mn_*x*_Fe_3−*x*_O_4_–DEA/MWCNT nanocomposites is dominated by dielectric loss, owing to increased electrical conductivity and interfacial polarization with the addition of Mn. We believe that our findings may shed light on the role of substitution elements in developing the radar absorption properties not only for Mn_*x*_Fe_3−*x*_O_4_–DEA/MWCNT nanocomposites, but also for other iron oxides/graphene-based nanocomposites, which further pave the way for their real implementation as RAMs.

## Conflicts of interest

There are no conflicts to declare.

## Supplementary Material
